# Nutritional and physiological responses to dietary phosphorus levels and phytase in pullets and laying hens

**DOI:** 10.1016/j.psj.2024.103886

**Published:** 2024-05-22

**Authors:** Jina Park, Yun-Ji Heo, Da-Hye Kim, Yoo Bhin Kim, Byung-Yeon Kwon, Ju-Yong Song, Kyung-Woo Lee

**Affiliations:** Department of Animal Science and Technology, Konkuk University, Seoul 05029, Republic of Korea

**Keywords:** gut health, laying hen, mineral balance, phytase, corticosterone

## Abstract

The objective of this study was to determine the effects of dietary available phosphorus (**P**) levels and dietary phytase added into the very low-P diet on the performance, mineral balance, odor emission, and stress responses in growing pullets and laying hens during 13 to 32 wk of age. One hundred sixty-eight pullets (Hy-Line Brown) were randomly assigned into 1 of 4 dietary treatments with 7 replicates of 6 birds each. Experimental diets were formulated to contain 3 graded P levels at 0.25, 0.35, and 0.45% during 13 to 15 wk (phase 1), 0.25, 0.35, and 0.45% during 16 to 18 wk (phase 2), and 0.20, 0.30, and 0.40% during 19 to 32 wk (phase 3). In addition, dietary phytase (500 FTU/kg matrix values) was added into the very low-P diets (0.20% during 13–15 wk, 0.25% during 16–18 wk, and 0.20% during 19–32 wk) to meet the nutritional adequacy with standard P diets. In all phases, decreasing dietary P levels did not affect (*P* > 0.05) growth, laying performance, and egg qualities. Decreasing dietary P levels linearly increased the relative duodenal and oviduct weights (*P* < 0.05), and quadratically increased the relative ovary weight in pullets (*P* = 0.016). Dietary phytase lowered (*P* = 0.021) the relative duodenal weight compared with the very low-P diet. Tibia breaking strength and tibia Mg contents in pullets were linearly lowered (*P* < 0.05) as dietary P levels decreased. Dietary phytase tended to increase (*P* = 0.091) tibia breaking strength and significantly increased (*P* = 0.025) tibia Mg content compared with the very low-P diet. Dietary P levels and dietary phytase affected (*P* < 0.05) ileal crypt depth and ileal villus height: crypt depth ratio in pullets. Decreasing dietary P levels linearly decreased (*P* < 0.01) crude fat digestibility and P excretion in both pullets and laying hens. Dietary phytase reversed (*P* < 0.05) the very low-P diet-mediated decrease of crude fat digestibility in pullets and laying hens. Dietary P levels and dietary phytase affected (*P* < 0.05) odor emission including ammonia in pullets and total volatile fatty acids in laying hens. Finally, lowering dietary P levels increased (*P* < 0.01) yolk corticosterone concentrations and the increased corticosterone concentration by the very low-P diet was reversed by dietary phytase. Collectively, our study shows that decreasing dietary P levels induced nutritional and physiological responses in pullets and laying hens and these P-mediated negative effects were mitigated by dietary phytase.

## INTRODUCTION

Phosphorus (**P**) is a mineral that is essential for the growth and development of poultry (Boling et al., 2000). It serves a multitude of functions, including bone development, skeletal mineralization, and the regulation of key enzymes involved in metabolism, as well as playing a role in various genomic and physiological processes (Abbasi et al., 2019). Most organic P found in cereal grains and meals used for poultry diets exists in the form of phytic acid and phytate representing 60 to 82% of total P (Ravindran et al., 1994; Ojha et al., 2019). However, endogenous enzymes responsible for breaking phytic acid are negligible (Abudabos, 2012) or almost absent in poultry resulting in decreased organic P availability (Raboy, 2009) and consequently a large amount of P is excreted (Hwangbo et al., 2007). P-rich excreta could be leached into groundwater that can increase the growth of algae and aquatic plants leading to water eutrophication (Ryden et al., 1974; Keshavarz, 2003; Knowlton et al., 2004; Ayoade et al., 2022). It is also known that environmental P stimulates organic matter degradation (Li et al., 2021) encouraging the production of odorous compound through microbial activity. In addition, dietary P is considered a nutritional factor affecting gut microbiota (Witzig et al., 2015; Ye et al., 2021) which could lead to odor production.

At this stage, the best strategy to overcome P excretion would be to lower dietary P levels in combination with exogenous phytase addition into the diets of chickens (Lin et al., 2017). It was shown that P excretion decreased in laying hens fed diets containing low available P levels without negative effect on laying performance and tibia characteristics (Keshavarz, 1986). Panda et al. (2005) reported that dietary phytase added into the low P diet increased the availability of phytate P and lowered P excretion in laying hens. While it is expected that a low P diet could affect the bone nutrition and metabolism pullets (Dijkslag et al., 2023), there is limited research on the effects of a low P diet on odorous compound emission of pullets and laying hens. Earlier studies Borda-Molina et al. (2016) and Roth et al. (2022) reported that dietary P affected gut microbiota which might affect gut health indicators as well as odor emission including fecal short chain fatty acids. Finally, although the low P diet could be utilized without affecting growth and laying performance in pullets and laying hens, it is not known whether it could be acted as a nutritional stressor triggering stress responses in laying hens. Wickramasuriya et al. (2022) indicated that gut microbiota and physiology are closely connected to affect brain activity including stress responses. However, the possibility of low P diet being the nutritional stressor has not been tested in chickens. Thus, the purpose of this study was to investigate the effects of dietary P levels on growth performance, mineral (i.e., P, calcium, nitrogen, magnesium) balance, yolk corticosterone, and odor emission in pullet and laying hens. In addition, we tested whether dietary phytase could counteract nutritional stress responses by the very low-P diet, if exists, in laying hens.

## MATERIALS AND METHODS

The experimental protocols were approved by the Institutional Animal Care and Use Committee at Konkuk University (KU21082).

### Birds, Diets, and Experimental Design

An experiment had 3 feeding phases from 13 to 16 wk (phase 1), 17 to 18 wk (phase 2), and 19 to 32 wk (phase 3). A total of one hundred sixty-eight 13-wk-old pullets (Hy-Line Brown) were housed in cages and randomly subjected to one of 4 dietary treatments with 7 replicates per treatment. Each cage (45 × 45 × 45 cm) housed 2 pullets and 3 adjacent 3 cages considered a replicate. Initial lighting program was used 10L:14D at 13 wk and the light exposure was gradually increased at 18 wk to reach 16L:8D at 24 wk per the breeders’ guideline (Hy-Line, 2018). The temperature and relative humidity in the experimental room was maintained at 21 ± 2°C and 60%.

Three P diets using Monocalcium phosphate (**MCP**) and limestone were formulated to contain 0.25, 0.35, and 0.45% during 13 to 15 wk (Table 1), 0.25, 0.35, and 0.45% during 16 to 18 wk (Table 2), and 0.20, 0.30, 0.40% during 19 to 32 wk (Table 3). The standard-P diet is considered to contain 0.45, 0.45, and 0.40% P in phases 1, 2, and 3, respectively. Dietary P levels were lowered by 1% point in all phases. Dietary phytase (500 FTU/kg; Axtra PHY GOLD, Danisco Animal Nutrition, DuPont Industrial Biosciences, Marlborough, UK) was supplemented into the very low-P diet (i.e., 0.25, 0.25, and 0.20% in phases 1, 2, and 3) allowing the nutrients and energy matrix values of phytase. The matrix value (61.46 kcal/kg ME, 5.33 g/kg CP, 0.23 g/kg Lys, 0.24 g/kg Met + Cys, 0.20 g/kg Thr, 0.03 g/kg Trp, 0.27 g/kg Val, 0.18 g/kg sodium, 1.98 g/kg calcium, and 1.86 g/kg available P of phytase 500 FTU) was applied in the phytase-supplemented diet to achieve the equal nutrient and energy specification of the standard-P diet. All diets were isocaloric, isonitrogenous, and equal calcium (**Ca**) levels. All experimental diets were provided in the form of a mesh. Feed and water were provided ad libitum during the experiment period.

**Table 1:** The ingredients and chemical composition of the experimental diets in phase 1 (developer diet; 13 – 15 wk), as-fed basis.

Item	Level of available phosphorus^1^			
	Very low-P	Low-P	Standard-P	Very low-P + phytase^2^
Ingredients				
Corn	72.74	72.74	72.74	71.50
Soybean meal, 47% CP	20.00	20.00	20.00	19.10
Corn gluten meal	1.50	1.50	1.50	1.50
Tallow	0.35	0.35	0.35	0.35
L-lysine HCl, 78.8%	0.10	0.10	0.10	0.10
DL-methionine, 99%	0.09	0.09	0.09	0.09
Cellulose, Solka Floc	1.47	1.22	0.98	4.13
Sodium bicarbonate	0.60	0.60	0.60	0.54
Monocalcium phosphate	0.80	1.25	1.70	0.90
Limestone	1.90	1.70	1.49	1.33
Vitamin premix^3^	0.20	0.20	0.20	0.20
Mineral premix^4^	0.25	0.25	0.25	0.25
Phytase^5^	-	-	-	0.01
Total	100	100	100	100
Calculated chemical composition				
AMEn^6^, kcal/kg	2850.00	2850.00	2850.00	2850.00
Crude protein	16.00	16.00	16.00	16.00
Calcium	0.90	0.90	0.90	0.90
Available phosphorus	0.25	0.35	0.45	0.45
Ca:Available Phosphorus ratio	3.6:1	2.6:1	2:1	2:1
Phytate phosphorus	0.21	0.24	0.24	0.21
Total phosphorus	0.46	0.59	0.69	0.66
Lysine	0.86	0.86	0.86	0.85
Methionine	0.36	0.36	0.36	0.36
Methionine + Cysteine	0.70	0.70	0.70	0.70
Threonine	0.60	0.60	0.60	0.60
Tryptophan	0.17	0.17	0.17	0.17
Analyzed value				
Dry matter	86.25	86.03	86.17	86.07
Crude protein	16.63	16.51	16.86	16.32
Ether extract	1.89	1.90	1.91	1.94
Calcium	0.93	0.91	0.97	0.91
Total phosphorus	0.51	0.67	0.75	0.56
Ash	5.94	6.17	5.95	5.86

1 Very low-P; 0.25% available P, Low-P; 0.35% available P, Standard-P; 0.45% available P, Very low-P + phytase; 0.25% available P + phytase 500 FTU.

2 This diet was formulated with nutritional matrix values by phytase (i.e., 61.46 kcal/kg ME, 5.33 g/kg CP, 0.23 g/kg Lys, 0.24 g/kg Met + Cys, 0.20 g/kg Thr, 0.03 g/kg Trp, 0.27 g/kg Val, 0.18 g/kg sodium, 1.98 g/kg Ca, and 1.86 g/kg available P).

3 Vitamin premix provided following nutrients per kg of diet: vitamin A as retinyl acetate, 20,000 IU; vitamin D_3_ as cholecalciferol, 4,600 IU; vitamin E as α-tocopheryl acetate, 40 mg; vitamin K_3_ as menadione nicotinamide bisulfite, 4 mg; vitamin B_1_ as thiamin dilaurylsulfate, 4 mg; vitamin B_2_ as riboflavin, 8 mg; vitamin B_6_ as pyridoxine hydrochloride, 6 mg; vitamin B_12_ as cyanocobalamin, 0.04 mg; biotin, 0.24 mg; _D_-pantothenic acid as _D_-pantothenic acid, 18.37 mg; niacin as nicotinamide, nicotinic acid, 60 mg, folic acid as folate, folic acid, 1.4 mg, butylated hydroxytoluene 0.25 mg.

4 Mineral premix provided following nutrients per kg of diet: Fe as iron sulfate, 70 mg; Mn as manganese sulfate, 80 mg; Zn as zinc oxide, 60 mg; Cu as copper sulfate, 8 mg; Co as cobalt sulfate, 0.13 mg; I as calcium iodate, 1 mg; Se as selenium yeast, 0.20 mg.

5 Phytase provided by Danisco Animal Nutrition, DuPont Industrial Biosciences, Marlborough, UK.

6 AMEn, nitrogen-corrected apparent metabolization energy, kcal/kg.

**Table 2:** The ingredients and chemical composition of the experimental diets in phase 2 (prelay diet; 16–18 wk), as-fed basis.

Item	Level of available phosphorus^1^			
	Very low-P	Low-P	Standard-P	Very low-P + phytase^2^
Ingredients				
Corn	64.86	64.86	64.86	67.80
Soybean meal, 47% CP	21.27	21.27	21.27	19.70
Corn gluten meal	1.50	1.50	1.50	1.50
Tallow	2.72	2.72	2.72	1.44
L-lysine HCl, 78.8%	0.10	0.10	0.10	0.10
DL-methionine, 99%	0.09	0.09	0.09	0.09
Cellulose, Solka Floc	0.50	0.28	0.03	0.98
Sodium bicarbonate	0.65	0.65	0.65	0.56
Monocalcium phosphate	0.80	1.25	1.70	0.90
Limestone	7.06	6.83	6.63	6.47
Vitamin premix^3^	0.20	0.20	0.20	0.20
Mineral premix^4^	0.25	0.25	0.25	0.25
Phytase^5^	-	-	-	0.01
Total	100	100	100	100
Calculated chemical composition				
AMEn^6^, kcal/kg	2850.00	2850.00	2850.00	2850.00
Crude protein	16.00	16.00	16.00	16.00
Calcium	2.70	2.70	2.70	2.70
Available phosphorus	0.25	0.35	0.45	0.45
Ca:Available Phosphorus ratio	10.8:1	7.7:1	6.0:1	6.0:1
Phytate phosphorus	0.21	0.21	0.21	0.21
Total phosphorus	0.46	0.56	0.66	0.66
Lysine	0.88	0.88	0.88	0.86
Methionine	0.36	0.36	0.36	0.36
Methionine + Cysteine	0.62	0.62	0.62	0.63
Threonine	0.60	0.60	0.60	0.60
Tryptophan	0.17	0.17	0.17	0.17
Analyzed value				
Dry matter	87.13	87.20	87.10	87.23
Crude protein	15.89	15.91	15.95	16.01
Ether extract	2.14	2.01	2.26	1.98
Calcium	2.52	2.66	2.59	2.54
Total phosphorus	0.46	0.59	0.68	0.48
Ash	10.66	10.43	10.75	10.12

1 Very low-P; 0.25% available P, Low-P; 0.35% available P, Standard-P; 0.45% available P, Very low-P + phytase; 0.25% available P + phytase 500 FTU.

2 This diet was formulated with nutritional matrix values by phytase (i.e., 61.46 kcal/kg ME, 5.33 g/kg CP, 0.23 g/kg Lys, 0.24 g/kg Met + Cys, 0.20 g/kg Thr, 0.03 g/kg Trp, 0.27 g/kg Val, 0.18 g/kg sodium, 1.98 g/kg Ca, and 1.86 g/kg available P).

3 Vitamin premix provided following nutrients per kg of diet: vitamin A as retinyl acetate, 20,000 IU; vitamin D_3_ as cholecalciferol, 4,600 IU; vitamin E as α-tocopheryl acetate, 40 mg; vitamin K_3_ as menadione nicotinamide bisulfite, 4 mg; vitamin B_1_ as thiamin dilaurylsulfate, 4 mg; vitamin B_2_ as riboflavin, 8 mg; vitamin B_6_ as pyridoxine hydrochloride, 6 mg; vitamin B_12_ as cyanocobalamin, 0.04 mg; biotin, 0.24 mg; _D_-pantothenic acid as _D_-pantothenic acid, 18.37 mg; niacin as nicotinamide, nicotinic acid, 60 mg, folic acid as folate, folic acid, 1.4 mg, butylated hydroxytoluene 0.25 mg.

4 Mineral premix provided following nutrients per kg of diet: Fe as iron sulfate, 70 mg; Mn as manganese sulfate, 80 mg; Zn as zinc oxide, 60 mg; Cu as copper sulfate, 8 mg; Co as cobalt sulfate, 0.13 mg; I as calcium iodate, 1 mg; Se as selenium yeast, 0.20 mg.

5 Phytase provided by Danisco Animal Nutrition, DuPont Industrial Biosciences, Marlborough, UK.

6 AMEn, nitrogen-corrected apparent metabolization energy, kcal/kg.

**Table 3:** The ingredients and chemical composition of the experimental diets in phase 3 (peak diet; 19–32 wk), as-fed basis.

Item	Level of available phosphorus^1^			
	Very low-P	Low-P	Standard-P	Very low-P + phytase^2^
Ingredients				
Corn	57.96	57.96	57.96	57.83
Soybean meal, 47% CP	16.52	16.52	16.52	21.76
Corn gluten meal	7.75	7.75	7.75	3.16
Soybean oil	3.00	3.00	3.00	3.00
L-lysine HCl, 78.8%	0.38	0.38	0.38	0.23
DL-methionine, 99%	0.11	0.11	0.11	0.13
L-threonine, 99%	0.08	0.08	0.08	0.06
Salt	0.16	0.16	0.16	0.16
Sodium bicarbonate	0.40	0.40	0.40	0.35
Choline chloride, 50%	0.06	0.06	0.06	0.06
Monocalcium phosphate	0.50	0.88	1.26	0.55
Limestone	11.78	11.60	11.43	11.14
Cellulose, Solka Floc	0.85	0.65	0.44	1.11
Vitamin premix^3^	0.20	0.20	0.20	0.20
Mineral premix^4^	0.25	0.25	0.25	0.25
Phytase^5^	-	-	-	0.01
Total	100	100	100	100
Calculated chemical composition				
AMEn^6^, kcal/kg	2800.00	2800.00	2800.00	2800.00
Crude protein	17.50	17.50	17.50	17.50
Calcium	4.29	4.29	4.29	4.29
Available phosphorus	0.20	0.30	0.40	0.40
Ca:Available Phosphorus ratio	21.4:1	14.3:1	10.7:1	10.7:1
Phytate phosphorus	0.19	0.19	0.19	0.19
Total phosphorus	0.39	0.49	0.59	0.39
Lysine	1.00	1.00	1.00	1.00
Methionine	0.42	0.42	0.42	0.42
Methionine + Cysteine	0.72	0.72	0.72	0.72
Threonine	0.70	0.70	0.70	0.70
Tryptophan	0.16	0.16	0.16	0.16
Analyzed value				
Dry matter	89.16	89.06	88.90	88.88
Crude protein	17.66	17.59	17.60	17.84
Ether extract	4.31	4.83	4.81	5.10
Calcium	4.28	4.25	4.29	3.70
Total phosphorus	0.38	0.48	0.60	0.38
Ash	17.06	16.33	15.71	14.89

1 Very low-P; 0.20% available P, Low-P; 0.30% available P, Standard-P; 0.40% available P, Very low-P + phytase; 0.20% available P + phytase 500 FTU.

2 This diet was formulated with nutritional matrix values by phytase (i.e., 61.46 kcal/kg ME, 5.33 g/kg CP, 0.23 g/kg Lys, 0.24 g/kg Met + Cys, 0.20 g/kg Thr, 0.03 g/kg Trp, 0.27 g/kg Val, 0.18 g/kg sodium, 1.98 g/kg Ca, and 1.86 g/kg available P).

3 Vitamin premix provided following nutrients per kg of diet: vitamin A as retinyl acetate, 20,000 IU; vitamin D_3_ as cholecalciferol, 4,600 IU; vitamin E as α-tocopheryl acetate, 40 mg; vitamin K_3_ as menadione nicotinamide bisulfite, 4 mg; vitamin B_1_ as thiamin dilaurylsulfate, 4 mg; vitamin B_2_ as riboflavin, 8 mg; vitamin B_6_ as pyridoxine hydrochloride, 6 mg; vitamin B_12_ as cyanocobalamin, 0.04 mg; biotin, 0.24 mg; _D_-pantothenic acid as _D_-pantothenic acid, 18.37 mg; niacin as nicotinamide, nicotinic acid, 60 mg, folic acid as folate, folic acid, 1.4 mg, butylated hydroxytoluene 0.25 mg.

4 Mineral premix provided following nutrients per kg of diet: Fe as iron sulfate, 70 mg; Mn as manganese sulfate, 80 mg; Zn as zinc oxide, 60 mg; Cu as copper sulfate, 8 mg; Co as cobalt sulfate, 0.13 mg; I as calcium iodate, 1 mg; Se as selenium yeast, 0.20 mg.

5 Phytase provided by Danisco Animal Nutrition, DuPont Industrial Biosciences, Marlborough, UK.

6 AMEn, nitrogen-corrected apparent metabolization energy, kcal/kg.

Body weight was measured at 13, 18, and 32 wk of age on a cage basis. Feed intake was recorded every 3 wk.

### Sample Collection

At 18 wk, one pullet per replicate was randomly selected and euthanized with an overdose of carbon dioxide for sampling blood. The serum samples were obtained after centrifugation at 200 × *g* for 15 min and stored at –20°C until analysis. Immediately after blood sampling, abdominal fat, liver, kidney, spleen, small intestine (duodenum, jejunum, ileum), bursa of Fabricius, ovary, oviduct, and tibia were collected, weighed, and expressed relative to body weight. Yellow follicles were recorded by counting only those over 0.5 cm in diameter. A 1-cm long mid-ileal segment were collected and fixed with 10% neutral buffered formalin solution for histological observations. Tibiae were obtained by manually removing the attached meat and cartilage. Left tibia was used for dry matter, ash, and strength, and right tibia was used to measure the concentrations of Ca, P, and magnesium (**Mg**). Physical tibia characteristics including width and length of tibia, breaking strength, and fat-free tibia weight were analyzed as described earlier (Heo et al., 2023). Villus height and crypt were observed in 7 well-oriented intact villi at 40 × magnification using a digital microscope (Olympus BX43, Tokyo, Japan) and photographed using a digital camera (eXcope T500, Olympous Tokyo, Japan) (Heo et al., 2023).

### Measurements of Calcium, P, Magnesium in Tibia and Serum Samples

The fat-freed tibiae were ashed at 600°C for 3 h to analyze Ca, P, and Mg contents using inductively coupled plasma optical emission spectrometer (ICP-OES; Avio 200, PerkinElmer Inc., Waltham, MA). All elements were detected at specific wavelengths (P, 213.617; Ca, 393.366; Mg 279.353 nm) to gain maximum signal intensity and minimum spectral overlap (Chung et al., 2013). Serum samples collected at 18 wk were analyzed for P, Ca, and Mg using an automatic blood chemical analyzer (Film Dry CHEM 7000i, Fuji Film, Tokyo, Japan).

### Retention and Excretion of Nutrients

At 18 and 32 wk, 2 birds per replicate were housed in metabolic cages to measure the retention and excretion of nutrients using total fecal collection method. Birds were allowed ad libitum to feed and water. Feed consumption was recorded, and fecal droppings were quantitatively collected twice a day during 3 consecutive days. After eliminating foreign substances (feathers, feed, etc.), the collected excreta were pooled per replicate and stored at –20°C until drying. Excreta samples were dried in a force-air-drying oven at 65°C for 72 h and ground for chemical analysis. Feed and excreta were analyzed for dry matter (DM; method 930.15), crude protein (CP; method 990.03), crude fat (method 920.39), and crude ash (method 942.05). In addition, feed and excreta samples were analyzed to determine Ca, P, and Mg using the ICP-OES (Avio 200, PerkinElmer Inc., Waltham, MA) at specific wavelengths of each element (P, 214.914; calcium, 317.933; Mg 279.079 nm). Nutrient digestibility and mineral balance were calculated based on the specified nutrient concentrations of the excreta voided and the feed consumed.

### Measurement of Odorous Compounds

At the end of 18 and 32 wk, freshly voided fecal samples were collected from 2 birds. Odor emission for carbon dioxide, ammonia, hydrogen sulfide, and trimethylamine using the modified flux chamber method and total volatile fatty acids (**VFA**) using gas chromatography were analyzed as described earlier (Lee et al., 2022b; Heo et al., 2023). Fecal VFA are considered odorous compounds (Jensen and Hansen, 2006; Le et al., 2008; Yang et al., 2016).

### Laying Performance and Egg Quality

During 19 to 32 of age, egg weight and egg production were recorded daily and used to calculate first egg production, 50% egg production, and egg mass. The percentage of soft and broken eggs was calculated as total number of soft and broken eggs per replicate divide by total number of eggs per replicate multiple by 100. The feed conversion ratio was calculated as feed intake divided by egg mass.

During the last 3 d at 30 wk of age, 6 intact eggs per replicate were collected for the egg quality measurement. Haugh unit, eggshell strength, yolk color score, and eggshell thickness (without shell membrane) were measured by a digital egg tester (DET6000, Nabel Co., Ltd., Kyoto, Japan). Yolk color was automatically graded on a scale of 1 to 26, with 1 being a very pale yellow and 16 being a dark orange. Eggshell color was measured by a shell color reflectometer (TSS QCR Technical Services, and Supplies, York, UK). Values closer to 0 indicate a darker color (black) while value closer to 81.8 indicate a lighter color (white).

### Measurement of Yolk Corticosterone

During the last 3 d at 30 wk of hen age, 3 intact eggs per replicate were randomly collected for corticosterone quantification in yolk samples. The separated yolks were pooled, homogenized, and mixed with an equal volume of ethanol. Then, the mixture was incubated at 37°C for 1 h, and subsequently centrifuged. The supernatants were analyzed with CORT ELISA kit (Enzo Life Science Inc, ADI-901-097, Farmingdale, NY) as previously described (Kozlowski and Bauman, 2009; Kim et al., 2021).

### Statistical Analysis

Three adjacent cages were considered as an experimental unit. All data were analyzed using Proc MIXED procedure of SAS (SAS Inst. Inc., Cary, NC). The results were presented as least squares means and pooled standard error of the means. Tukey test was employed to determine means and differences among treatments. In addition, the linear and quadratic effect of 3 P levels in diets were analyzed using the polynomial contrasts. Significant differences were preset at *P* < 0.05.

## RESULTS

### Growth Performance and Organ Development

Experiment performed well and only one hen died in the moderate-P group during 19 to 32 wk of age. Lowering P levels and dietary phytase added into the very low-P diet did not affect final body weight, body weight gain and feed intake in pullets (Table 4). At 18 wk, none of dietary P levels and phytase addition affected the relative weights of abdominal fat, liver, kidney, spleen, and bursa of Fabricius (Table 5). Lowering dietary P levels linearly increased the relative duodenal weight, but dietary phytase lowered (*P* < 0.05) it compared with the very low-P diet-fed pullets. Relative jejunal and ileal weights were not affected by dietary P levels or dietary phytase (Table 5). Lowering dietary P levels quadratically increased the relative ovary weight (*P* < 0.05) and linearly increased the relative oviduct weight (*P* < 0.05). Dietary phytase tended to increase the relative ovary weight compared with the very low-P diet and significantly increased it compared with the standard-P diet. The number of large yellow follicles was not affected by dietary P levels or phytase addition.

**Table 4:** Effect of dietary available phosphorus levels and phytase (500 FTU/kg) on the growth performance in pullets.^1^

Item	Level of available phosphorus^2^				SEM^3^	*P*-value		
	Very low-P	Low-P	Standard-P	Very low-P + phytase		ANOVA	L^4^	Q^4^
Initial BW^5^, kg/hen	1.23	1.26	1.15	1.23	0.04	0.178	0.124	0.164
Final BW^5^, kg/hen	1.71	1.69	1.63	1.68	0.04	0.651	0.283	0.702
BWG^6^, kg/hen	0.47	0.44	0.49	0.44	0.03	0.606	0.792	0.283
ADFI^7^, g/day/hen	71.9	73.3	72.4	73.5	0.78	0.417	0.517	0.122

1 All means are average of 7 replicates per treatment.

2 Very low-P; 0.25% available P, Low-P; 0.35% available P, Standard-P; 0.45% available P, Very low-P + phytase; 0.25% available P + phytase 500 FTU.

3 SEM, standard error of the mean.

4 L and Q *P*-value for 3 P levels.

5 BW, body weight.

6 BWG, body weight gain.

7 ADFI, average daily feed intake.

**Table 5:** Effect of dietary available phosphorus levels and phytase (500 FTU/kg) on the relative organ weight (g/100 g of live body weight) and the number of follicles in pullets.^1^

Item	Level of available phosphorus^2^				SEM^3^	*P*-value		
	Very low-P	Low-P	Standard-P	Very low-P + phytase		ANOVA	L^4^	Q^4^
Abdominal fat	2.38	2.67	2.03	2.28	0.32	0.573	0.394	0.192
Liver	1.65	1.51	1.59	1.76	0.07	0.078	0.523	0.170
Kidney	0.573	0.547	0.588	0.639	0.040	0.430	0.817	0.531
Spleen	0.214	0.197	0.232	0.219	0.016	0.491	0.454	0.233
Duodenum	0.478^a^	0.404^ab^	0.365^b^	0.374^b^	0.026	0.021	0.010	0.607
Jejunum	0.782	0.848	0.770	0.791	0.043	0.593	0.865	0.243
Ileum	0.667	0.671	0.601	0.640	0.023	0.211	0.095	0.263
Bursa of Fabricius	0.120	0.136	0.144	0.114	0.025	0.828	0.497	0.887
Ovary	0.080^ab^	0.093^ab^	0.066^b^	0.102^a^	0.008	0.016	0.210	0.044
Oviduct	0.306	0.219	0.101	0.259	0.064	0.173	0.016	0.815
The number of large yellow follicles	0.571	0.429	0.000	0.429	0.355	0.695	0.232	0.725

a,b Means within a row without a common superscript letter differ (*P* < 0.05). Expressed as grams per 100 g body weight

1 All means are average of 7 replicates per treatment.

2 Very low-P; 0.25% available P, Low-P; 0.35% available P, Standard-P; 0.45% available P, Very low-P + phytase; 0.25% available P + phytase 500 FTU.

3 SEM, standard error of the mean.

4 L and Q *P*-value for 3 P levels.

### Characteristics and Mineral Composition of Tibia

Tibia characteristics (i.e., fresh weight, width and length of tibia, fat-free weight) except for breaking strength were not altered by dietary P levels or dietary phytase supplementation (Table 6). Tibia breaking strength was decreased as dietary P levels increased. Dietary phytase numerically increased tibia breaking strength by 26.3% compared with the very low-P diet. Dietary P levels or phytase did not affect Ca and P contents in tibia. However, Mg contents in tibia linearly decreased as dietary P levels lowered. In addition, dietary phytase significantly increased tibia Mg contents compared with the very low-P diet (Table 6). Dietary P levels or dietary phytase did not affect the concentration of Ca, P, and Mg in serum samples of pullets (Table 7).

**Table 6:** Effect of dietary available phosphorus levels and phytase (500 FTU/kg) on tibia characteristics in pullets.^1^

Item	Level of available phosphorus^2^				SEM^3^	*P*-value		
	Very low-P	Low-P	Standard-P	Very low-P + phytase		ANOVA	L^4^	Q^4^
Fresh weight^5^, g	0.550	0.583	0.575	0.590	0.016	0.365	0.281	0.316
Width, cm	0.891	0.911	0.894	0.906	0.022	0.891	0.911	0.499
Length, cm	8.97	9.01	9.03	9.04	0.07	0.885	0.505	0.904
Breaking strength, kgf	18.6	21.1	23.3	23.5	1.5	0.091	0.028	0.889
Dry matter, %	67.9	67.9	64.5	63.7	1.8	0.215	0.202	0.466
Ash/fat-free dry matter, %	41.3	37.5	39.7	39.3	1.4	0.311	0.483	0.126
Composition, %								
Calcium	38.0	38.7	38.5	40.2	0.9	0.512	0.768	0.687
Phosphorus	18.7	19.2	19.4	19.5	0.4	0.633	0.405	0.753
Magnesium	0.563^b^	0.608^ab^	0.626^ab^	0.631^a^	0.015	0.025	0.026	0.520

a,b Means within a row without a common superscript letter differ (*P* < 0.05).

1 All means are average of 7 replicates per treatment.

2 Very low-P; 0.25% available P, Low-P; 0.35% available P, Standard-P; 0.45% available P, Very low-P + phytase; 0.25% available P + phytase 500 FTU.

3 SEM, standard error of the mean.

4 L and Q *P*-value for 3 P levels.

5 Fresh weight was expressed as grams per 100 g body weight.

**Table 7:** Effect of dietary available phosphorus levels and phytase (500 FTU/kg) on mineral concentrations in serum samples of pullets.^1^

Item	Level of available phosphorus^2^				SEM^3^	*P*-value		
	Very low-P	Low-P	Standard-P	Very low-P + phytase		ANOVA	L^4^	Q^4^
Calcium, mg/dL	11.84	11.39	11.46	11.91	0.52	0.854	0.617	0.692
Phosphorus, mg/dL	9.03	10.06	10.06	10.20	0.67	0.805	0.607	0.597
Magnesium, mg/dL	1.43	1.36	1.54	1.70	0.16	0.470	0.598	0.346

^a,b^Means within a row without a common superscript letter differ (*P* < 0.05).

1 All means are average of 7 replicates per treatment.

2 Very low-P; 0.25% available P, Low-P; 0.35% available P, Standard-P; 0.45% available P, Very low-P + phytase; 0.25% available P + phytase 500 FTU.

3 SEM, standard error of the mean.

4 L and Q *P*-value for 3 P levels.

### Ileal Morphology

Villus height was not affected by dietary P levels or phytase supplementation although pullets fed on the phytase-added diet had highest (*P* = 0.072) villus height (Table 8). Decreasing P levels in diets linearly lowered crypt depth, but increased villus height: crypt depth ratio. Dietary phytase significantly increased crypt depth and tended to decrease villus height: crypt depth ratio compared with pullets fed on the very low-P diet (Table 8).

**Table 8:** Effect of dietary available phosphorus levels and phytase (500 FTU/kg) on ileal morphology in pullets.^1^

Item	Level of available phosphorus^2^				SEM^3^	*P*-value		
	Very low-P	Low-P	Standard-P	Very low-P + phytase		ANOVA	L^4^	Q^4^
Villus height, µm	1,090	1,043	1,002	1,169	42.7	0.072	0.142	0.947
Crypt depth, µm	144^b^	175^ab^	179^a^	186^a^	10.5	0.006	0.027	0.188
Villus height: Crypt depth^5^	7.70^a^	6.24^ab^	6.17^b^	6.33^ab^	0.40	0.005	0.011	0.176

a,b Means within a row without a common superscript letter differ (*P* < 0.05).

1 All means are average of 7 replicates per treatment.

2 Very low-P; 0.25% available P, Low-P; 0.35% available P, Standard-P; 0.45% available P, Very low-P + phytase; 0.25% available P + phytase 500 FTU.

3 SEM, standard error of the mean.

4 L and Q *P*-value for 3 P levels.

5 Villus to Crypt, villus height divides Crypt depth.

### Nutrient Digestibility and Mineral Balance

At 18 wk, dietary P levels or phytase addition did not affect the amount of excreta voided and the digestibilities of DM, CP and crude ash in pullets. However, crude fat digestibility linearly decreased (*P* < 0.001) as dietary P levels lowered (Table 9). In addition, pullets fed on the phytase-added diet improved crude fat digestibility compared with the very low-P diet. Dietary P levels or phytase addition did not affect the retained nitrogen (**N**), Ca, P, Mg and the excreted N, Ca, and Mg in pullets. However, the excreted P linearly decreased as dietary P levels lowered (*P* < 0.001). Dietary phytase did not affect the excreted P compared with the very low-P diet (*P* < 0.001). At 32 wk (Table 10), patterns on the amount of total excreta voided, nutrient digestibility and mineral balance were similar as noted in pullets assayed at 18 wk. Decreasing dietary P levels linearly lowered fat digestibility and P excretion in laying hens. Dietary phytase added into the very low-P diet increased fat digestibility, but did not affect the P excretion compared with the very low-P diet.

**Table 9:** Effect of dietary available phosphorus levels and phytase (500 FTU/kg) on the nutrient retention and excretion in excreta of pullets.^1^

Item	Level of available phosphorus^2^				SEM^3^	*P*-value		
	Very low-P	Low-P	Standard-P	Very low-P + phytase		ANOVA	L^4^	Q^4^
Total excreta, g/d/bird	86.9	85.2	89.5	95.9	5.1	0.473	0.700	0.614
Nutrient digestibility, %								
Dry matter	72.6	71.1	72.2	72.3	0.7	0.472	0.691	0.170
Crude protein	55.8	51.4	53.1	60.6	3.0	0.170	0.547	0.439
Crude fat	66.1^b^	62.7^b^	83.2^a^	80.7^a^	1.3	< 0.001	< 0.001	0.081
Crude ash	19.3	15.9	21.0	20.6	2.0	0.272	0.519	0.077
Mineral balance, g/d/bird								
Retention								
Nitrogen	1.14	0.99	1.16	1.28	0.09	0.151	0.862	0.132
Calcium	0.254	0.205	0.236	0.212	0.061	0.568	0.844	0.213
Phosphorus	0.124	0.131	0.134	0.159	0.016	0.477	0.635	0.914
Magnesium	0.404	0.693	0.537	0.682	0.096	0.134	0.394	0.110
Excretion								
Nitrogen	0.881	0.962	1.017	0.821	0.076	0.293	0.258	0.902
Calcium	1.63	1.83	1.80	1.59	0.10	0.228	0.271	0.388
Phosphorus	0.219^c^	0.305^b^	0.399^a^	0.228^c^	0.018	< 0.001	< 0.001	0.876
Magnesium	1.52	1.30	1.28	1.32	0.12	0.494	0.215	0.535

a,b Means within a row without a common superscript letter differ (*P* < 0.05).

1 All means are average of 7 replicates per treatment.

2 Very low-P; 0.25% available P, Low-P; 0.35% available P, Standard-P; 0.45% available P, Very low-P + phytase; 0.25% available P + phytase 500 FTU.

3 SEM, standard error of the mean.

4 L and Q *P*-value for 3 P levels.

**Table 10:** Effect of dietary available phosphorus levels and phytase (500 FTU/kg) on the nutrient retention and excretion in excreta of laying hens.^1^

Item	Level of available phosphorus^2^				SEM^3^	*P*-value		
	Very low-P	Low-P	Standard-P	Very low-P + phytase		ANOVA	L^4^	Q^4^
Total excreta, g/d/bird	86.0	85.2	90.4	89.3	4.4	0.801	0.490	0.587
Nutrient digestibility, %								
Dry matter	74.8	76.8	72.8	72.2	1.8	0.276	0.464	0.223
Crude protein	56.5	55.9	40.2	45.7	5.4	0.117	0.077	0.327
Crude fat	84.2^b^	83.4^b^	92.2^a^	93.1^a^	1.8	0.001	0.014	0.077
Crude ash	54.7	55.9	40.5	42.5	5.7	0.144	0.129	0.297
Mineral balance, g/d/bird								
Retention								
Nitrogen	2.29	2.63	2.15	2.31	0.14	0.121	0.496	0.083
Calcium	2.13	2.30	2.16	1.67	0.18	0.114	0.931	0.543
Phosphorus	0.112	0.164	0.113	0.090	0.031	0.402	0.982	0.214
Magnesium	0.74	1.29	1.38	1.23	0.19	0.099	0.058	0.363
Excretion								
Nitrogen	1.23	1.14	1.31	1.13	0.11	0.660	0.683	0.396
Calcium	2.05	2.18	2.32	2.19	0.14	0.637	0.238	0.990
Phosphorus	0.274^b^	0.362^b^	0.511^a^	0.316^b^	0.026	< 0.001	< 0.001	0.366
Magnesium	1.93	1.55	1.65	1.81	0.17	0.421	0.302	0.294

a,b Means within a row without a common superscript letter differ (*P* < 0.05).

1 All means are average of 7 replicates per treatment.

2 Very low-P; 0.20% available P, Low-P; 0.30% available P, Standard-P; 0.40% available P, Very low-P + phytase; 0.20% available P + phytase 500 FTU.

3 SEM, standard error of the mean.

4 L and Q *P*-value for 3 P levels.

### Odor Emission

Among the odor compounds tested, fecal ammonia concentration linearly decreased (*P* = 0.057) as dietary P levels lowered in pullets (Table 11). Dietary phytase added into the very low-P diet significantly increased fecal ammonia concentration compared with the very low-P diet. At 32 wk, none of dietary treatments affected odor compounds including carbon dioxide, ammonia, hydrogen sulfide, and trimethylamine in fecal samples. However, decreasing dietary P levels linearly increased total VFA concentration, and dietary phytase tended to decrease it by 9.6% compared with the very low-P diet (Table 11).

**Table 11:** Effect of dietary available phosphorus levels and phytase (500 FTU/kg) on the odorous compound emission (ppm) in excreta of pullets (18 wk) and laying hens (32 wk).^1^

Item	Level of available phosphorus^2^				SEM^3^	*P*-value		
	Very low-P	Low-P	Standard-P	Very low-P + phytase		ANOVA	L^4^	Q^4^
Wk 18								
Carbon dioxide, ppm	750	680	690	676	74.0	0.883	0.581	0.670
Ammonia, ppm	45.5^b^	59.4^b^	62.8^ab^	82.8^a^	5.8	0.003	0.057	0.474
Hydrogen sulfide, ppm	0.75	0.85	1.40	1.03	0.17	0.608	0.100	0.518
Trimethylamine, ppm	159	163	170	104	19.0	0.095	0.714	0.961
Total VFA, mM/g	97.0	98.4	98.1	97.2	0.6	0.879	0.495	0.595
Wk 32								
Carbon dioxide, ppm	368	342	348	364	20.17	0.767	0.473	0.507
Ammonia, ppm	4.40	4.20	4.40	5.00	1.10	0.960	1.000	0.865
Hydrogen sulfide, ppm	N.D	N.D	N.D	N.D	N.D	N.D	N.D	N.D
Trimethylamine, ppm	8.60	7.40	8.00	7.00	1.47	0.876	0.782	0.632
Total VFA, mM/g	98.1	92.1	89.6	88.7	2.8	0.059	0.029	0.832

a,b Means within a row without a common superscript letter differ (*P* < 0.05).

1 All means are average of 7 replicates per treatment.

2 Very low-P; 0.25% available P, Low-P; 0.35% available P, Standard-P; 0.45% available P, Very low-P + phytase; 0.25% available P + phytase 500 FTU in pullets, Very low-P; 0.2% available P, Low-P; 0.30% available P, Standard-P; 0.40% available P, Very low-P + phytase; 0.20% available P + phytase 500 FTU in laying hens.

3 SEM, standard error of the mean.

4 L and Q *P*-value for 3 P levels.

### Laying Performance, Egg Quality, and Yolk Corticosterone

The effects of dietary P levels and phytase did not affect laying performance including feed intake, egg production, egg weight, egg mass, FCR, soft and broken eggs, and age at 50% egg production (Table 12). However, decreasing dietary P levels quadratically increased final body weight and age at first egg production (Table 12). Dietary P levels or phytase did not affect egg qualities including Haugh unit, eggshell strength, eggshell thickness, and eggshell color. Decreasing dietary P levels linearly increased yolk color and dietary phytase decreased it compared with the very low-P diet-fed laying hens (Table 13). The effect of dietary P levels and dietary phytase on stress hormone was studied to see if the low P diet would affect stress responses and if so dietary phytase could reverse the low P-induced stress response in laying hens. Concentration of yolk corticosterone was linearly and quadratically increased as dietary P levels lowered. Of interest, dietary phytase added into the very low-P diet significantly lowered yolk corticosterone compared with the lowest P diet-fed laying hens (Table 13).

**Table 12:** Effect of dietary available phosphorus levels and phytase (500 FTU/kg) on the laying performance in laying hens.^1^

Item	Level of available phosphorus^2^				SEM^3^	*P*-value		
	Very low-P	Low-P	Standard-P	Very low-P + phytase		ANOVA	L^4^	Q^4^
Growth performance								
Initial BW^5^, kg/hen	1.71	1.69	1.63	1.68	0.04	0.651	0.283	0.702
Final BW^5^, kg/hen	1.90	1.99	1.86	1.92	0.04	0.186	0.520	0.029
ADFI^6^, g/day/hen	102	103	100	103	1.4	0.382	0.302	0.206
Egg performance								
Egg production, %	80.6	78.4	78.4	78.7	2.4	0.899	0.404	0.672
Egg weight, g/egg	59.5	60.4	59.6	59.7	0.8	0.863	0.914	0.449
Egg mass, g/d	48.0	47.4	46.6	46.9	1.6	0.931	0.489	0.966
FCR^7^, g:g	2.14	2.19	2.14	2.22	0.08	0.848	0.996	0.491
Soft and broken egg, %	0.02	0.06	0.06	0.05	0.02	0.547	0.241	0.493
Age of egg production								
First of egg production, day	142	147	142	144	1.5	0.160	0.940	0.015
50% of egg production, day	147	150	149	149	1.7	0.801	0.448	0.511

1 All means are average of 7 replicates per treatment.

2 Very low-P; 0.20% available P, Low-P; 0.30% available P, Standard-P; 0.40% available P, Very low-P + phytase; 0.20% available P + phytase 500 FTU.

3 SEM, standard error of the mean.

4 L and Q *p*-value for 3 P levels.

5 BW, body weight.

6 ADFI, average daily feed intake.

7 FCR, feed conversion ratio.

**Table 13:** The effect of available phosphorus levels on egg quality and yolk corticosterone concentrations in laying hens at 30 wk of age.^1^

	Level of available phosphorus^2^				SEM^3^	*P*-value		
Item	Very low-P	Low-P	Standard-P	Very low-P + phytase		ANOVA	L^4^	Q^4^
Haugh unit	95.4	95.5	94.6	95.7	0.7	0.758	0.510	0.645
Eggshell strength, kgf	5.12	5.07	5.09	4.95	0.15	0.873	0.888	0.837
Eggshell thickness, mm	0.391	0.396	0.387	0.389	0.007	0.855	0.655	0.441
Eggshell color^5^	25.0	25.5	26.8	24.1	0.9	0.247	0.218	0.778
Yolk color, RCF^6^	7.08^a^	6.76^ab^	6.52^b^	5.44^c^	0.13	< 0.001	0.004	0.781
Corticosterone in yolk, pg/g	150^a^	55^b^	47^b^	55^b^	12	< 0.001	< 0.001	0.011

a,b, Means within a row without a common superscript letter differ (*P* < 0.05).

1 All means are average of 7 replicates per treatment.

2 Very low-P; 0.20% available P, Low-P; 0.30% available P, Standard-P; 0.40% available P, Very low-P + phytase; 0.20% available P + phytase 500 FTU.

3 SEM, standard error of the mean.

4 L and Q *P*-value for 3 P levels.

5 Eggshell color was measured using a shell color reflectometer to provide a reflectance value from 0% (black) to 81.8% (white).

6 RCF, Roche yolk color fan.

## DISCUSSION

Phosphorus, as an essential mineral, is the third most expensive nutrient and is added at the ranges from 0.45% to 0.48% for growing pullets and from 0.36% to 0.49% for laying hens (Hy-Line, 2018). Nonetheless, attempts to lower dietary P levels in the diets for laying hens has been implemented due to the negative aspect of the excreted P into the environment (Boling et al., 2000; Panda et al., 2005; Hwangbo et al., 2007; Jing et al., 2021). Although nutritional strategy with low P diets to maintain laying performance and egg qualities has been well studied, it is not known whether low P diet would affect nutritional and physiological responses including odor emission, stress response, and mineral balance, and whether dietary phytase added into the very low-P diet would counteract the negative aspect, if any, of very low-P diet in growing pullets and laying hens.

Our study shows that lowering P levels in the diets of growing pullets from 0.45% to 0.25% did not affect growth performance (i.e., body weight and feed intake) of pullets as reported elsewhere (Keshavarz, 1986; Boling et al., 2000; Jing et al., 2018) indicating that pullets could adapt to change in dietary P levels (Jing et al., 2018). Nonetheless, lowering P levels in the diet of pullets increased the relative organ weights (i.e., duodenum, ovary, and oviduct). Whether the increased reproductive organ weight (i.e., ovary and oviduct) in pullets fed the P-deficient diets is related to the compensatory responses to lower P levels needs to be clarified. As P is absorbed at duodenum and jejunum, increased duodenal weight seen in pullets fed the very low-P diet is likely to maximize the P absorption. Indeed, earlier study (Akyurek et al., 2011) has reported that broiler fed a low P diet exhibited an increase in the relative length of the duodenum. Additionally, other studies have indicated that dietary P absorption can be enhanced by upregulating the expression of the Na-P IIb cotransporter in the duodenum (Nie et al., 2013; Pongmanee et al., 2020). However, further study is required to validate the latter indication. Furthermore, our finding that pullets fed the phytase-added, very low-P diet had similar duodenal weight compared with the standard-P diet-fed group indicates the phytase-mediated P release mitigating hypertrophy or hyperplasia of duodenum in the very low-P diet-fed pullets.

The tibia characteristics are considered essential factors in evaluating P absorption in poultry, and a deficiency of P in the feed can lead to bone disorders in poultry (Bai et al., 2004; Huttunen et al., 2006; Ren et al., 2023). In addition, the tibia characteristics *vs.* growth performance are considered more sensitive indicator for P deficiency (Selle and Ravindran, 2007). Indeed, our study shows that tibia breaking strength became weakier with decreasing P levels in pullets. We noted that the lowering P diet-induced decrease in tibia breaking strength is closely associated with the lowered concentrations of Mg, but not Ca or P, in tibia. Also, dietary supplementation of phytase into the very low-P diet was able to increase the tibia strength as well as Mg contents in tibia compared with the very low-P diet. Thus, it is likely that low available P concentrations at gut levels mediated by low P diets may inhibit the Mg bioavailability leading to poor bone mineralization. In line with our findings, standard *vs*. deficient P diets (Viveros et al., 2002; Han et al., 2009) or dietary phytase (Um et al., 1999; Singh et al., 2013; Mansoori and Modirsanei, 2015) have been known to increase the tibia strength and/or the Mg bioavailability in tibia.

Serum Ca and P concentrations might be considered the important indicators of nutritional status for pullets (Yang et al., 2009). Nonetheless, even in a Ca and P-deficient diet, Ca and P from pullet bones are utilized to maintain normal Ca and P homeostasis in the serum, thereby supporting normal physiological functions of muscles, nerves, and other tissues (Yang et al., 2009). In addition, the lack of dietary P levels on the mineral concentrations in serum samples noted in this study is consistent with the results of other studies (Cheng et al., 2020; Jing et al., 2021).

Intestinal development and morphology, including villus height, crypt depth, and the villus height: crypt depth ratio, are important for efficient absorption of energy and nutrients (Mathivanan et al., 2006; Chiang et al., 2010; Heo et al., 2023). Earlier studies reported that a low P diet reduced the ileal villus height and the villus height: crypt depth ratio in broiler chickens, and that the P-mediated effect was recovered when phytase was added into the low P diet (Emami et al., 2013; Nari et al., 2020). However, our study showed that decreasing dietary P levels did not affect villus height but linearly decreased, thus leading to the increase in villus height: crypt depth ratio. Although lower crypt depth and higher villus height: crypt depth ratio are often considered beneficial gut health indicator in chickens (Qaisrani et al., 2014; Peng et al., 2016), our finding needs an careful interpretation whether lowering dietary P levels indeed improved gut environment compared with pullets fed on the standard-P or phytase-added diet.

The results of this study showed that crude fat digestibility decreased linearly as the P levels decreased and dietary phytase reversed the low P-mediated decrease in fat digestibility in both pullets and laying hens. Previous studies have reported that Ca-phytate complex with fatty acids in the gut lumen to form insoluble soaps, thereby lowering fat digestibility (Leeson, 1993; Ravindran et al., 2000). Camden et al. (2001) and Liu et al. (2010) reported that this problem could be overcome by adding phytase in the feed. However, as all experimental diets had equal Ca and phytate levels, it is not likely whether insoluble soaps were excessively formed only in the very low-P diet. Thus, it might be likely that lowering dietary P levels could impair either fat digestion or absorption processes. Earlier studies (Li et al., 2016) reported that low P diets inhibited pancreatic lipase activity. Thus, if this is the case, lowering dietary P levels could have lowered lipase activity leading to poor fat micelle formation for efficient absorption. In any events, this needs to be further addressed.

As expected, decreasing dietary P levels linearly lowered the P excretion in both growing pullets and laying hens as reported elsewhere (Paik et al., 2000; Liebert et al., 2005; Jing et al., 2018). This study clearly demonstrates that lowering dietary P levels can be efficient means to lower P excretion in a dose-dependent manner. Of interest, the retained Mg was lowest, but the excreted Mg was highest in the very low-P diet-fed pullets and laying hens. Thus, it is likely that the very low-P diet could impair the Mg bioavailability by lowering Mg retention (and increasing Mg excretion) leading to lower tibia Mg contents in the very low-P diet-fed pullets seen in this study.

Odorous compound emissions from excreta are serious environmental pollutants associated with agricultural systems (Wang et al., 2021). As odorous compounds including VFAs in excreta are mainly produced through microbial fermentation on undigested nutrient (Le et al., 2005; Aarnink et al., 2007) and dietary P levels are known to affect gut microbiome (Borda-Molina et al., 2016; Roth et al., 2022), it is hypothesized that odor emission would be affected by dietary P levels or dietary phytase. Lowering dietary P levels lowered ammonia concentrations in pullets but increased total VFAs in laying hens. In addition, adding phytase into the diet of pullets, but not of laying hens, increased the very low-P diet-mediated decrease in ammonia concentration. Our study indicates that dietary P levels could affect gut microbiome as reported earlier (Li et al., 2022), but this effect was different at pullets and laying hens.

During laying period, no significant effect of dietary P levels and dietary phytase was noted in laying performance and egg qualities although quadratic effect by dietary P levels was noted in final body weight, age at first egg production, and yolk color. The quadratic effect by dietary P levels on final body weight might be a consequence of altered feed intake, but the difference in body weight between treatment groups was not of magnitude. In addition, the quadratic effect by dietary P levels on age at first egg production might be due to the heavier body weight in laying hens fed the low-P diet. Finally, when phytase was added into the very low-P diet in this study, yolk color was significantly lowered. This effect was secondary to the reduction of corn gluten meal in the phytase-added, very low-P diet compared with the very low-P diet as reported elsewhere (Galobart et al., 2004). In addition, yolk color was getting paler as dietary P levels decreased in this study. In line with our finding, deficient- *vs*. standard-P diets produced paler egg yolks in laying hens (Englmaierova et al., 2015).

In this study, we tested whether nutritional P deficiency would affect stress responses in laying hens as the abrupted gut homeostasis is known to trigger stress responses leading to increased secretion of stress hormones (Mumma et al., 2006; Rostagno, 2020). Indeed, we found that yolk corticosterone concentrations were increased with decreasing dietary P levels and dietary low P-induced increase in yolk corticosterone was reversed by dietary phytase. Thus, our study suggests that lowering P levels activate the gut-brain axis triggering stress responses in laying hens. In line with our finding, it has been reported that laying hens raised under suboptimal conditions such as high stocking density and heat stress increased corticosterone in blood and yolk (Lee et al., 2022a; Kim and Lee, 2023). In addition, Heo et al. (2023) reported that higher CP diet increased yolk corticosterone in laying hens. The results of this study suggest that low P diets may cause stress responses in laying hens, but the P-induced stress can be mitigated by dietary phytase. As far as we know, this is the first report to show that dietary P deficiency causes nutritional stress responses that can be relieved by dietary phytase.

It was concluded that reducing dietary P levels affected the relative duodenal weight, tibia breaking strength, ileal morphology (crypt depth and villus height: crypt depth ratio), fat digestibility, P excretion, and fecal ammonia production in pullets or laying hens. Dietary phytase lowered the relative duodenal weight and villus height: crypt depth ration, but increased tibia breaking strength, tibia Mg contents, crypt depth, fat digestion and fecal ammonia production compared with the very low-P diet. Finally, yolk corticosterone concentrations were increased by decreasing dietary P levels and were reversed back to normal by dietary phytase. Collectively, our study shows that dietary P levels could affect nutritional and physiological responses of pullets and laying hens and those harmful responses could be mitigated by dietary phytase.

## DISCLOSURES

The authors declare no conflicts of interest.
